# Sexual Dysfunction Improved in Heroin-Dependent Men after Methadone Maintenance Treatment in Tianjin, China

**DOI:** 10.1371/journal.pone.0088289

**Published:** 2014-02-10

**Authors:** Minying Zhang, Huifang Zhang, Cynthia X. Shi, Jennifer M. McGoogan, Baohua Zhang, Linglong Zhao, Mianzhi Zhang, Keming Rou, Zunyou Wu

**Affiliations:** 1 School of Medicine, Nankai University, Tianjin, China; 2 National Center for AIDS/STD Control and Prevention, Chinese Center for Disease Control and Prevention, Beijing, China; 3 Ankang Hospital, Tianjin, China; 4 Public Security Hospital, Tianjin, China; Kaohsiung Chang Gung Memorial Hospital, Taiwan

## Abstract

**Objective:**

To investigate whether methadone maintenance treatment (MMT) is correlated with sexual dysfunction in heroin-dependent men and to determine the prevalence and risk factors of sexual dysfunction among men on MMT.

**Methods:**

The study included a retrospective survey and a cross-sectional survey which contained interviews of 293 men who are currently engaged in MMT. The results of the two surveys were compared. For a subset of 43 participants, radioimmunoassay was additionally conducted using retrospective and prospective blood samples to test the levels of plasma testosterone and luteinizing hormone. Other study evaluations were the International Index of Erectile Function (IIEF-15), and Self-rating Depression Scale.

**Results:**

Sexual dysfunction in all five IIEF-15 domains (erectile function, orgasmic function, sexual desire, intercourse satisfaction, and overall satisfaction) was strongly associated with long-term use of heroin. A decrease in the severity of sexual dysfunction was associated with MMT initiation. Erectile dysfunction, lack of sexual desire, inability to orgasm, and lack of intercourse satisfaction were significantly correlated with increasing age of the participants. Methadone dose and duration of methadone treatment were not found to be associated with sexual dysfunction. The level of plasma testosterone significantly declined during methadone treatment, but results from multivariate analysis indicated low levels of testosterone were not the main cause of sexual dysfunction. No correlation between reported depression status and sexual function was found.

**Conclusions:**

While high levels of sexual dysfunction were reported by heroin-dependent men in our study before and after MMT initiation, MMT appears to be correlated with improved sexual function in the population of the study.

## Introduction

The association between sexual dysfunction and use of opioids has been described extensively [1]–[3]. Previous studies found that sexual desire was enhanced among drug users during the initial six months after starting drug use. However, heroin users reported gradually decreasing and disappearing sexual interest. Palha and Esteves found that 72% of men and 65% of women who used heroin for more than six months reported decreasing satisfaction with their sexual behavior [4].

Using methadone maintenance treatment (MMT) to treat opioid dependence began in the United States in 1964, and it has been shown to be an effective intervention in controlling drug use among heroin addicts [5], [6]. Positive effects of methadone maintenance include reduced frequency of opioid use[7], reduced frequency of criminal behavior [8], reduced HIV transmission [9], mortality reduction [10], and improved employment status [11].

Numerous previous studies have reported varying prevalence of sexual dysfunction among individuals in MMT [12]–[20]. As early as in 1972, Cushman et al. and Brown et al. found that a respective 30% and 14% of their male patients on MMT reported erectile dysfunction [12], [20]. Studies have not established whether methadone itself or whether other factors during MMT are the cause of sexual dysfunction. Decreases in plasma testosterone, methadone dose, age, and depression have been suggested as the contributing factors to sexual dysfunction, but previous studies have not consistently demonstrated these mechanisms [15], [16], [18], [20].

Many studies show higher rates of sexual dysfunction in men on MMT than in the general population [15]–[18]. Few studies have examined whether the sexual dysfunction increases or decreases among individuals who are receiving MMT compared with that in MMT-naive heroin-dependent individuals. Hanbury et al. found 33% of 50 men reported sexual dysfunction shortly after initiating MMT, most of whom had experienced similar difficulties when using heroin [13].

Most studies in China on MMT patients are limited to characteristics of sexual behavior, such as the frequency of sexual activity, the number of sexual partners, and condom use [21], [22]. Few studies have examined the male sexual function change during MMT. This article aims to assess the level of sexual dysfunction in heroin-dependent men before and after MMT initiation, to determine the prevalence and change of sexual dysfunction, and to explore the influencing factors by assessing behavioral, psychological and social factors.

## Methods

### Study design and setting

This cross-sectional study was conducted in Ankang Hospital in Tianjin, China from September 2011 to November 2011. All participants were recruited in the MMT clinic of Ankang hospital, which is the only setting for providing MMT in Tianjin. At the time of the study, the clinic was providing MMT to approximately 600 heroin-dependent individuals, of which 398 were men.

### Participants

Participants were recruited in the MMT clinic of Ankang Hospital. Subjects were eligible for this study if they 1) were men over 18 years old, 2) had been engaged in MMT for at least one month, and 3) had at least one sexual encounter while they were dependent on heroin within the past two years. Subjects were then excluded if their frequency of sexual intercourse was reduced for reasons other than sexual dysfunction, such as having an HIV infection or being separated from their partners.

### Interviews and measures

A questionnaire was used to record the information of the participants. The questionnaire included items on demographic characteristics, drug use details (such as history of drug use, daily dose of drugs, injecting drug or not, and needle sharing), methadone treatment status (such as the time of receiving MMT and methadone dose), and sexual behaviors (such as number of sex partners, frequency of sexual intercourse and condom use within 3 months before initiating MMT and the interview). Participants were asked to retrospectively describe their sexual behavior before and after initiating MMT. The interviews were conducted individually in a private location by the researcher themselves. The questionnaires were completed anonymous and the scales were filled out by the participants themselves in a private location.

Participants were also asked to complete the Chinese version of International Index of Erectile Function (IIEF-15) [23], and the Zung Self-rating Depression Scale (SDS) [24] after the interview. The IIEF is a brief, reliable, and multidimensional scale for assessing sexual function in men in both research and clinical trials [25].The IIEF-15 is an extensively validated questionnaire covering five domains of male sexual function: erectile function (six items), orgasmic function (two items), sexual desire (two items), intercourse satisfaction (three items), and overall satisfaction (two items), with the total of 15 items. Maximum scores for the IIEF-15 are as follows: erectile function (30), orgasmic function (10), sexual desire (10), intercourse satisfaction (15), and overall satisfaction (10). Higher scores indicate less sexual dysfunction. The responses are calculated to provide a severity rating for each domain: no dysfunction, mild dysfunction, mild-moderate dysfunction, moderate dysfunction, and severe dysfunction [26]. The SDS is an internationally validated measure of 20 items designed to assess the severity of depression symptoms [27]. SDS scores are divided into four ratings: normal, mildly depressed, moderately depressed, and severely depressed. The IIEF-15 and SDS were designed to collect the information of one month before initiating MMT and one month before the interview.

### Sample collection and laboratory testing

All newly-enrolled MMT clients are required to provide a blood sample, which is tested immediately for human immunodeficiency virus (HIV), hepatitis C virus and syphilis infection, with the remaining sample being reserved in long-term storage for later study. At the start of the study, 43 participants’ blood samples were in a well-preserved state in the clinic laboratory. The 43 participants were identified, and we collected a second blood sample from these participants after their study interview and questionnaires were completed. The retrospective and prospective samples were measured to determine the levels of plasma testosterone and luteinizing hormone (LH) before and after initiating MMT. Testosterone and luteinizing hormone concentrations were determined using an immunoradiometric assay (Tianjin Union Medical Technology Group Co., Ltd, in Tianjin, China). The sensitivities for testosterone and luteinizing hormone were 0.1 ng/dl and 0.4 mIU/ml. The range for intra-assay coefficients of variation (CV) for testosterone assayed in duplicate was 5.95% and the inter-assay CV was 7.27%. The range for intra-assay CV for luteinizing hormone assayed in duplicate was7.64% and the inter-assay CV was 9.16%.

### Statistical Analysis

Categorical variables are presented as number and percent and were compared using a chi-square test. Continuous variables are presented as mean and standard deviation (SD).Wilcoxon signed ranks test was used to compare the difference of sexual dysfunction before and after MMT initiation according to the number of participants who got different levels of dysfunction classified by IIEF-15. Univariate analysis was used to analyze factors influencing different domains of sexual dysfunction. Based on the univariate analysis results, multivariate logistic regression (forward inclusion and backward stepwise) models were constructed with statistically significant independent variables to calculate adjusted odds ratios (OR) for the exploration of factors associated with sexual function in men before and after MMT initiation. All confidence intervals (CIs) presented are 95% CIs, and p-values less than 0.05 were deemed statistically significant. All of the questionnaires were complete in the study. Statistical analysis was performed using Stata software (Version 12, College Station, TX, USA).

### Ethical issues

The study design and procedures were approved by the Institutional Review Board of Nankai University. Participants were fully informed about the aim and procedure of the study and written informed consent was received from all participants. Each participant could obtain ten times of free methadone treatment as a reward for his participation and they were informed about the reward after the study. The interview and the scales were conducted anonymously and all personal identifiers were removed from the final dataset to preserve participant privacy.

## Results

Among the 398 men attending the Ankang Hospital MMT clinic in Tianjin, China at the start of our study, a total of 368 were eligible for participation in the study. Among them, 23 men declined to participate and a further 52 men were excluded because they reported purposely reduced the frequency of sexual intercourse for reasons other than sexual dysfunction. These reasons included HIV-infection (n = 4), being separated from their sexual partners (n = 21), and not having a regular sexual partner (n = 27). A total of 293 men were included in this study.

### Demographics, behavioral, and treatment characteristics

The mean age of the participants was 39.7 ± 8.7 years. Most participants were 40 to 49 years old (42.7%), of Han ethnicity (93.9%), and married (44.4%). Participants who had received a middle school education or above accounted for 85.3%, and more than half of the total participants (57.3%) were currently unemployed. As shown in Table 1.Before receiving MMT, the participants had used drugs for a median length of 10 years with their first heroin use at a mean age of 27.8 ± 7.7. 83.6% of participants regularly used heroin by injection, 9.0% of them reporting sharing syringes with friends and other individuals.

**Table 1:** Demographic and self-reported heroin use characteristics of participants.

Characteristics	Participants N (%)
**1.demographic characteristics**	
**Age (years)**	
20–29	52 (17.7)
30–39	81 (27.6)
40–49	125 (42.7)
50–59	35 (12.0)
**Mean age**	39.7±8.7
**Ethnicity**	
Han	275 (93.9)
Others	18 (6.1)
**Education level**	
Under primary school	43 (14.7)
Middle school	165 (56.3)
High school*	77 (26.3)
College or higher	8 (2.7)
**Marital status**	
Single	72 (24.6)
Married/Cohabitating	155 (52.9)
Divorced/separated/widowed	66 (22.5)
**Employed**	
Yes	125(2.7)
No	168(7.3)
**2.history of drug use**	
Age at first heroin use (mean ± SD)	27.8±7.7
Median of drug use (years)	10
**Regular drug use method**	
Injection	245 (83.6)
No injection	48 (16.4)
**Sharing of syringes**	
Yes	22 (9.0)
No	223 (91.0)
**3.Methadone maintenance treatment (MMT)**	
**Duration of MMT(months)**	
1∼12	100 (34.1)
12∼18	193 (65.9)
**Methadone dose (ml)**	
∼50	38 (13.0)
50∼110	248 (84.6)
110∼160	7 (2.4)
**Positive in urine test**	
Yes	65 (22.2)
No	228 (77.8)
Overall	293 (100.0)

* High school category includes senior high school, secondary vocational school, or technical school.

The duration of methadone treatment of the subjects ranged from one month to 1.5 years (median = 1.1 years). 65.9% of the subjects had participated in MMT for more than one year. The range of daily methadone dose for most participants (84.6%) was between 50 ml and 110 ml, and 22.2% had received at least one heroin-positive urine test.

All study participants reported having experienced sexual activity before and only heterosexual experiences. During heroin dependence and methadone treatment, most participants reported sexual activity less than 4 times per month, accounting for 40.3% and 35.5% respectively (Table 2). After heroin dependence developed, there was no significant difference in sexual activity frequency before and after initiating MMT. During regular heroin use and methadone treatment, the majority of subjects had only one sexual partner. The proportion of participants having multiple sexual partners after MMT initiation was 9.9%, which was significantly lower than before MMT initiation (19.5%) (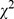 = 10.68, P<0.05).

**Table 2:** Self-reported sexual behavior characteristics of participants before and after MMT.

	During heroin use	During MMT		
Characteristics	(Before MMT) N (%)	N (%)	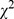	P
**Frequency of sex (times/week)** *				
0	105 (35.8)	87 (29.7)	2.510	0.113
< 1	118 (40.3)	104 (35.5)	1.421	0.233
1	39 (13.3)	56 (19.1)	3.631	0.057
2–5	28 (9.6)	40 (13.7)	2.396	0.122
≥6	3 (1.0)	6 (2.0)	0.451	0.502
**Number of sexual partners** *				
0	74 (25.2)	77 (26.3)	0.080	0.777
1	162 (55.3)	187 (63.8)	4.428	0.035
≥2	57 (19.5)	29 (9.9)	10.684	0.001
Overall	293 (100.0)	293 (100.0)		

MMT: methadone maintenance treatment.

* Frequency of sex and number of sexual partners (male or female) behaviors asked within three months before initiating MMT and the interview, respectively. Participants who had been engaged in MMT less than 3 months were asked within the duration of MMT.

### Depression, hormone levels characteristics

Based on the SDS scores, a high prevalence of depression was found during both heroin dependence and MMT. According to the scores, the psychological condition of the participants was classified into two categories: no depression (score<50) and depression (score≥50). During heroin dependence, 94.2% of the participants reported suffering from depression, which decreased to 29.4% of participants during MMT. The severity of depression of the clients was significantly improved after the receiving of MMT (*χ^2^* = 4.794, P<0.001), though average scores indicated that depression severity remained high.

The laboratory assessments of 43 participants’ plasma testosterone and luteinizing hormone showed that plasma testosterone levels decreased significantly from 343.49±126.41μg/dl to 284.53±91.36μg/dl after participants initiating MMT (t = –2.849, P = 0.007). The average luteinizing hormone level was 4.02±3.72μg/dl before MMT and 3.42±2.48μg/dl after MMT, and showed no significant changes (t = –0.887, P = 0.38). No association was found between the changes in plasma testosterone or luteinizing hormone levels and methadone treatment duration or methadone dose.

And the sub-sample of the 43 participants were not significantlydifferent from the other subjects in the study in age (t = –0.89, P = 0.37), duration of MMT (Z = –0.86, P = 0.45) and methadone dose (Z = –0.81, P = 0.42).

### Sexual function characteristic

The outcomes of the IIEF-15 over the five domains are summarized in Table 3.The median of the total score after receiving MMT was 33.0, which was significantly higher than that before MMT (median = 0) (Z = –10.456, P<0.0001). According to the scores, the severity of sexual dysfunction in each domain was classified into five categories: no sexual dysfunction (SD), mild, mild to moderate SD, moderate SD, and severe SD. Before MMT, 65.2% of the participants reported severe erectile dysfunction, 70.7% reported severe orgasm dysfunction, 71.7% reported severe lack of sexual desire, 69.6% reported severe lack of intercourse satisfaction, and 70.0% reported severe lack of overall satisfaction. However, after MMT, the prevalence of severe erectile dysfunction, orgasm dysfunction, lack of sexual desire, lack of intercourse satisfaction and lack of overall satisfaction was decreased to 37.2%, 39.3%, 43.0%, 39.6% and 41.0%, respectively. Although a high proportion of participants rated their sexual dysfunction as severe both before and after initiating methadone treatment, a significant number of subjects reported improvement after receiving MMT.

**Table 3:** Self-reported sexual dysfunction before and after MMT initiation.

	Before MMT	After MMT		
IIEF-15 Domain*	initiation N(%)	initiation N(%)	*Z* †	P-value†
**Erectile dysfunction(score range)**			–11.53	<0.0001
Severe (0–6)	191 (65.2)	109 (37.2)		
Moderate (7–12)	23 (7.9)	24 (8.2)		
Moderate to minor (13–18)	42 (14.3)	73 (24.9)		
Minor (19–24)	22 (7.5)	50 (17.1)		
No (25–30)	15 (5.1)	37 (12.6)		
**Inability to orgasm**			–9.12	<0.0001
Severe (0–2)	207 (70.6)	117 (39.9)		
Moderate (3–4)	27 (9.2)	46 (15.7)		
Moderate to minor (5–6)	26 (8.9)	64 (21.8)		
Minor (7–8)	22 (7.5)	42 (14.3)		
No (9–10)	11 (3.8)	24 (8.2)		
**Lack of sexual desire**			–7.16	<0.0001
Severe (0–2)	210 (71.7)	126 (43.0)		
Moderate (3–4)	38 (13.0)	54 (18.4)		
Moderate to minor (5–6)	32 (10.9)	67 (22.9)		
Minor (7–8)	10 (3.4)	35 (11.9)		
No (9–10)	3 (1.0)	11 (3.8)		
**Lack of intercourse satisfaction**			–8.10	<0.0001
Severe (0–3)	204 (69.6)	116 (39.6)		
Moderate (4–6)	39 (13.3)	56 (19.1)		
Moderate to minor (7–9)	35 (11.9)	77 (26.3)		
Minor (10–12)	13 (4.4)	37 (12.6)		
No (13–15)	2 (0.7)	7 (2.4)		
**Lack of overall satisfaction**			–9.49	<0.0001
Severe (0–2)	205 (70.0)	120 (41.0)		
Moderate (3–4)	30 (10.2)	38 (13.0)		
Moderate to minor (5–6)	32 (10.9)	59 (20.1)		
Minor (7–8)	22 (7.5)	63 (21.5)		
No (9–10)	4 (1.4)	13 (4.4)		

MMT: methadone maintenance treatment.

* IIEF-15 covering five domains of male sexual function: erectile function, orgasmic function, sexual desire, intercourse satisfaction and overall satisfaction.

† Wilcoxon signed ranks test and p-values generated using Wilcoxon signed ranks test.

### Factors associated with sexual dysfunction while dependent on heroin and during MMT

Under univariate analysis, older age and longer time of heroin use were found to be strong risk factors for sexual dysfunction both before initiating MMT and after MMT. Before the clients began MMT, erectile dysfunction (OR = 1.08, 95%CI: 1.04–1.13), inability to orgasm (OR = 1.09, 95%CI: 1.04–1.14), lack of sexual desire (OR = 1.13, 95%CI: 1.05–1.22), and lack of overall satisfaction (OR = 1.06, 95%CI: 1.01–1.11) showed an association with increasing age of the clients. Increased length of heroin use correlated with erectile dysfunction (OR = 1.09, 95%CI: 1.02–1.17), lack of sexual desire (OR = 1.22, 95%CI: 1.07–1.40), and lack of overall satisfaction (OR = 1.11, 95%CI: 1.02–1.22). Moreover, having a regular sexual partner (wife or girlfriend) not using heroin was also significantly associated with sexual dysfunction: erectile dysfunction (OR = 4.46, 95%CI: 1.58–12.60), inability to orgasm (OR = 7.22, 95%CI: 2.70–19.34), lack of sexual desire (OR = 5.78, 95%CI: 1.36–24.64), lack of intercourse satisfaction (OR = 5.49, 95%CI: 1.30–23.18), and lack of overall satisfaction (OR = 4.69, 95%CI: 1.47–14.98). During methadone treatment, erectile dysfunction (OR = 1.05, 95%CI: 1.02–1.09), inability to orgasm (OR = 1.05, 95%CI: 1.01–1.09), lack of sexual desire (OR = 1.06, 95%CI: 1.02–1.10), and lack of intercourse satisfaction (OR = 1.06, 95%CI: 1.02–1.11) were significantly associated with increasing age of the clients. Scores of sexual dysfunction in all five domains were also correlated with increased length of heroin use: erectile dysfunction (OR = 1.09, 95%CI: 1.03–1.15), inability to orgasm (OR = 1.08, 95%CI: 1.01–1.15), lack of sexual desire (OR = 1.11, 95%CI: 1.03–1.18), lack of intercourse satisfaction (OR = 1.08, 95%CI: 1.01–1.16), and lack of overall satisfaction (OR = 1.08, 95%CI: 1.02–1.15). Methadone dose and duration of methadone treatment were not correlated with sexual dysfunction.

Results of the multivariate analysis (Table 4 and Table 5) showed that risk factors for sexual dysfunction differed before and after initiating MMT. In this analysis, the outcomes of sexual dysfunction were divided into binary outcomes: severe dysfunction and moderate or below. Before MMT, older age, injecting heroin and having a regular sexual partner who not used heroin were the risk factors of erectile dysfunction and inability to orgasm. The dysfunction of lack of sexual desire was only associated with increasing age of clients on multivariate analysis. In addition, lack of intercourse satisfaction and overall satisfaction showed a significant correlation with injecting heroin and having a regular sexual partner not using heroin (Table 4). However, after MMT, erectile dysfunction, inability to orgasm, and lack of intercourse satisfaction were significantly associated only with older age of the clients. Lack of sexual desire showed a direct correlation with older age and increased length of heroin use. Overall severity of dysfunction was associated with the increased length of heroin use on multivariate analysis (Table 5).

**Table 4:** Multivariate analysis of factors associated with sexual dysfunction before MMT.

	Before MMT				
Factors OR(95%CI) *	Erectile dysfunction	Inability to orgasm	Lack of Sexual desire	Lack of intercourse satisfaction	Lack of overall satisfaction
Age	1.07	1.08	1.13	–	1.05
	(1.02–1.13)	(1.01–1.15)	(1.02–1.26)		(0.98–1.11)
Injection	5.88	6.02	–	7.35	8.77
	(1.82–19.23)	(1.62–22.22)		(1.76–30.30)	(2.48–31.25)
Having a	3.41	10.03	4.44	6.40	4.43
regular	(1.07–10.89)	(2.86–35.16)	(0.74–26.80)	(1.35–30.38)	(1.21–16.21)
partner not					
using heroin					

The reference category is moderate or less.

MMT: methadone maintenance treatment, OR: odds ratio, CI: 95% confidence interval.

* ORs and CIs were generated using logistic regression.

–: The variables were not statistically significant in the univariate analysis.

**Table 5:** Multivariate analysis of factors associated with sexual dysfunction after MMT.

Factors OR(95%CI) *	After MMT				
	Erectile dysfunction	Inability to orgasm	Lack of sexual desire	Lack of intercourse satisfaction	Lack of overall satisfaction
Age	1.04	1.05	1.06	1.05	–
	(1.01–1.08)	(1.01–1.09)	(1.02–1.10)	(1.00–1.10)	
Duration of	1.06	1.07	1.11	1.05	1.08
heroin use	(0.98–1.13)	(0.83–1.45)	(1.03–1.18)	(0.96–1.14)	(1.02–1.15)

The reference category is moderate or less.

MMT: methadone maintenance treatment, OR: odds ratio, CI: 95% confidence interval.

* ORs and CIs were generated using logistic regression.

–: The variables were not statistically significant in the univariate analysis.

## Discussion

Men in MMT have serious problems with sexual function in all five IIEF-15 domains (erectile function, sexual desire, orgasm, intercourse satisfaction, overall satisfaction). However, participants’ sexual function significantly improved after initiating methadone treatment, suggesting that MMT can relieve symptoms of sexual dysfunction in heroin-dependent men.

The research literature has noted high rates of sexual dysfunction in heroin user and MMT patient populations. Our study found 37.2% of participants in MMT had severe erectile dysfunction, which was higher than reported in other studies. Quaglio et al. reported 16% men receiving methadone and buprenorphine treatment in Italy suffered from severe erectile dysfunction [28].

Testosterone plays a crucial role in the generation of male sex desire and the maintenance of sexual function. Physiological studies indicate that depression of testosterone secretion due to heroin use may contribute to sexual dysfunction [20], [29]. Heroin use is thought to inhibit gonadotropin-releasing hormone production, which decreases the release of luteinizing hormone and subsequently reduces testosterone production. Heroin use can also promote the secretion of prolactin, which induces negative feedback on the release of luteinizing hormone and consequently, on the secretion of testosterone. Our study results demonstrated the plasma testosterone level in MMT-naive heroin users was lower than in the general population and further decreased in men receiving MMT. However, sexual function of the subjects was significantly improved after initiating MMT, which indicated that low levels of testosterone are not directly correlated with sexual dysfunction, which has been noted elsewhere [18]. While other studies have concluded that endocrinal causes are responsible only in a small number of cases of sexual dysfunction in the general population [30], it is unclear whether decreases in testosterone concentration contributes to sexual dysfunction among men in MMT [15], [31].

In this study, sexual dysfunction was not associated with methadone dose. In contrast, Crowley et al. reported that high doses of methadone had significantly reduced the frequency of male sexual behavior [32], and Brown et al. found methadone dose level had a significant correlation with increased orgasm dysfunction [20]. Our conflicting finding may be due to several reasons. The study site was at the only methadone clinic in Tianjin, and the majority of participants were characterized by long-term heroin use and large daily dose. A substantial proportion of subjects reported severe sexual dysfunction before initiating methadone treatment, and 87.0% of the subjects received more than 50 ml of methadone daily. Thus, the subjects in our study possessed a degree of specificity which would contribute to some results of the study.

Our results also indicated no association between methadone dose and levels of plasma testosterone and luteinizing hormone. In prior studies, no correlation has been found between small to moderate doses of methadone and plasma testosterone levels [14], [16], [33], [34], which is consistent with our findings. Further research is suggested to explore whether MMT dose has an effect on sexual function.

Previous epidemiological studies found that psychological and behavioral factors in heroin users and methadone-maintained population were associated with sexual dysfunction [18], [28], [35]. Quaglio’ study indicated that sexual dysfunction associated with depression scores in the SDS scale [28]. This study found that male participants reported varying degrees of depression both before and after receiving methadone treatment. However, we did not find a direct correlation between the status of depression and sexual function.

Studies in Europe and Australia have noted that subjects with regular sexual partners were less likely to experience sexual dysfunction [18], [28]. Having a sexual partner with a history of drug use and not having a regular sexual partner were associated with a greater likelihood of sexual dysfunction [18], [28]. In our study, participants with regular sexual partners who did not use heroin had a higher frequency of sexual dysfunction than participants without regular sexual partners or had a regular partner with a history of heroin use. These findings contradict the European and Australian studies [18], [28], perhaps due to cultural differences. Most of the participants in our study with spouses having no history of heroin use had been separated from their wives for a long time, and these participants reported no sexual relations with their wives. In contrast, couples who were both heroin users may have been more harmonious because they could understand each other better.

There were several limitations to our study. Firstly, we found that a proportion of the participants in MMT still used heroin intermittently during methadone treatment based on the results of regularly scheduled urine testing, although they would be excluded from MMT. Therefore, we are unable to account for the concurrent effect of active heroin use on male sexual function in a limited number of participants. Secondly, another limitation of the study is recall bias due to the retrospective study design. The participants’ duration of MMT ranged from one month to one year and a half, and 65.87% of subjects had participated in MMT more than one year. In our study, the participants needed to recall the sexual function status of one month before they initiated MMT. So there may be bias in the recalling symptoms of sexual dysfunction prior to initiating MMT, which would influence the result of this study. Additionally, the study site, which is the only methadone maintenance clinic in Tianjin, was established about two years prior to the start of the study. The majority of patients in the clinic were severely dependent on heroin. Many patients presented with a history of long-term heroin use and received a high daily dose of methadone. These factors may contribute to a higher frequency of sexual dysfunction among our study participants than other drug users and MMT patients.

The clinical implication of this study is that male sexual dysfunction is not caused by MMT. On the contrary, sexual dysfunction symptoms are significantly alleviated compared prior to receiving MMT. Future research should explore the specific mechanisms causing this phenomenon and the impact of long-term MMT on male sexual function.
